# Carnivore Contact: A Species Fracture Zone Delineated Amongst Genetically Structured North American Marten Populations (*Martes americana* and *Martes caurina*)

**DOI:** 10.3389/fgene.2020.00735

**Published:** 2020-07-13

**Authors:** Michael Lucid, Sam Cushman, Lacy Robinson, Andrea Kortello, Doris Hausleitner, Garth Mowat, Shannon Ehlers, Sara Gillespie, Leona K. Svancara, Jack Sullivan, Andrew Rankin, David Paetkau

**Affiliations:** ^1^Idaho Department of Fish and Game, Coeur d’Alene, ID, United States; ^2^US Forest Service, Rocky Mountain Research Station, Flagstaff, AZ, United States; ^3^Rainforest Ecological, Sandpoint, ID, United States; ^4^Grylloblatta Ecological Consulting, Nelson, BC, Canada; ^5^Seepanee Ecological Consulting, Nelson, BC, Canada; ^6^British Columbia Ministry of the Environment, Nelson, BC, Canada; ^7^Kootenai Tribe of Idaho, Bonners Ferry, ID, United States; ^8^Wildlife Genetics International, Nelson, BC, Canada; ^9^Department of Biological Sciences, University of Idaho, Moscow, ID, United States

**Keywords:** effective population size, gene flow, genetic structure, *Martes americana*, *Martes caurina*, spatial genetic distance, taxonomy

## Abstract

North American martens are forest dependent, influenced by human activity, and climate vulnerable. They have long been managed and harvested throughout their range as the American marten (*Martes americana*). Recent work has expanded evidence for the original description of two species in North America — *M. americana* and the Pacific Coast marten, *M. caurina* — but the geographic boundary between these groups has not been described in detail. From 2010 to 2016 we deployed 734 multi-taxa winter bait stations across a 53,474 km^2^ study area spanning seven mountain ranges within the anticipated contact zone along the border of Canada and the United States. We collected marten hair samples and developed genotypes for 15 polymorphic microsatellite loci for 235 individuals, and 493 base-pair sequences of the mtDNA gene *COI* for 175 of those individuals. Both nuclear and mitochondrial genetic structure identified a sharp break across the Clark Fork Valley, United States with *M. americana* and *M. caurina* occurring north and south of the break, respectively. We estimated global effective population size (*N*_*e*_) for each mountain range, clinal genetic neighborhood sizes (*NS*), calculated observed (*H*_*o*_) and expected (*H*_*e*_) heterozygosity, fixation index (*F*_*ST*__)_, and clinal measures of allelic richness (*Ar*), *H*_*o*_, and inbreeding coefficient (*F*_*IS*_). Despite substantial genetic structure, we detected hybridization along the fracture zone with both contemporary (nuclear DNA) and historic (mtDNA) gene flow. Marten populations in our study area are highly structured and the break across the fracture zone being the largest documented in North America (*F*_*ST*_ range 0.21–0.34, mean = 0.27). With the exception of the Coeur d’Alene Mountains, marten were well distributed across higher elevation portions of our sampling area. Clinal *NS* values were variable suggesting substantial heterogeneity in marten density and movement. For both *M. americana* and *M. caurina*, elevationaly dependent gene flow and high genetic population structure suggest that connectivity corridors will be important to ensuring long-term population persistence. Our study is an example of how a combination of global and clinal molecular data analyses can provide important information for natural resource management.

## Introduction

North American marten (*Martes* spp.) are a group for which genetic data are changing the understanding of how populations and lineages are structured across large landscapes (e.g., Colella et al., 2019) and provide an important example of how genetic data can inform wildlife management. Marten are actively harvested and managed throughout North America and may be sensitive to forest management (Kirk and Zielinski, 2009; Cushman et al., 2011; Moriarty et al., 2016), human recreation (Slauson et al., 2017), and climate change (Cushman et al., 2011; Wasserman et al., 2012a).

Originally classified as two species — the American (*Martes americana*) and Pacific Coast (*Martes caurina*) marten (Merriam, 1890) — further morphometrics resulted in the assignment of *M. caurina* as a subspecies of *M. americana* (Wright, 1946). Although some authors continued to refer to two separate groups of marten in North America (Dawson and Cook, 2012), marten were subsequently considered, and managed, under the single species umbrella of *M. americana* (Colella et al., 2019). Recent molecular work has supported the original morphologically based two species classification of *M. caurina* and *M. americana* (Stone et al., 2002; Small et al., 2003; Dawson et al., 2017). Analysis of widely spaced samples from western North America supports the longstanding notion (Wright, 1946) that there is a broad area of contact in the Inland Pacific Northwest including portions of British Columbia, Idaho, Montana, and Washington (Colella et al., 2019).

Lacking from the picture, but crucial to wildlife management, is more detailed sampling to delineate where each lineage occurs and the level of genetic structure and gene flow between and amongst those lineages. Although obtaining the data necessary to calculate these metrics can be challenging (Lindenmayer and Likens, 2010), the inclusion of multiple species in field surveys is becoming a cost effective way to increase the efficiency of field efforts because well-designed multi-species inventory techniques allow for simultaneous data collection for many species (Tucker and Simmons, 2009; Robinson et al., 2017; Lucid et al., 2018a).

From 2010 to 2016 we deployed 734 multi-taxa winter bait stations across a 53,474 km^2^ study area within the known marten contact zone (Colella et al., 2019) from which we collected mammal hair samples. We used marten hair samples from those collections to develop this study for which our objectives were to (1) identify the location of the fracture zone between lineages, (2) measure genetic structure within and between lineages, (3) calculate clinal and global effective population sizes for mountain ranges, and (4) consolidate that information into conservation and management recommendations.

## Materials and Methods

### Study Area

**FIGURE 1:**
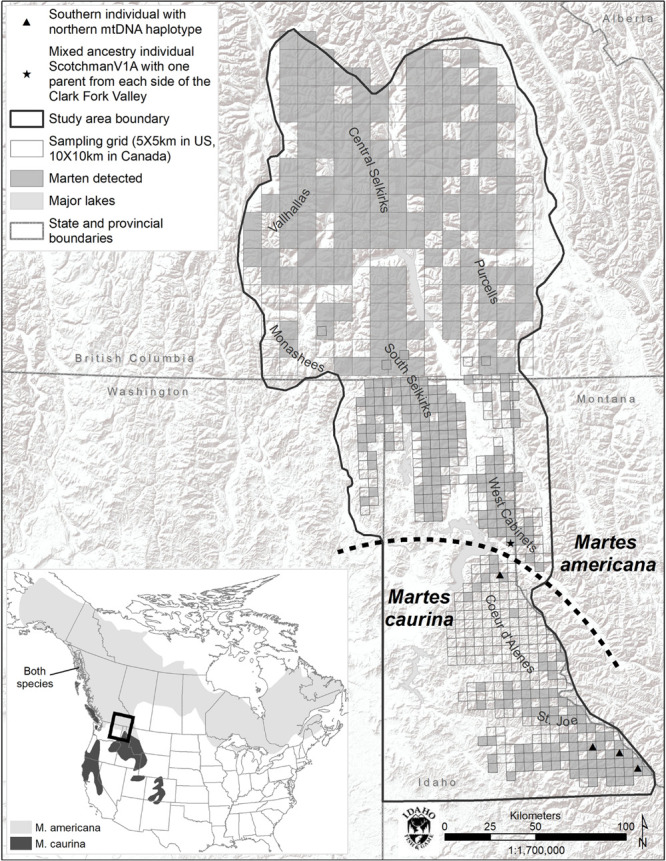
Study area map and species range map (inset). Marten detections include DNA, remote camera (US), and track (BC) detections.

The study area consisted of a 53,474 km^2^ area containing portions of British Columbia, Idaho, Montana, and Washington (Figure 1). It comprised portions of the Coeur d’Alene (CDA), Purcell, Saint Joe (Joe), Central Selkirk, South Selkirk, West Cabinet, and Valhalla Mountain ranges. Mountain ranges are generally intersected by wide, agriculturally dominated, low elevation valleys. Elevations range from 300 m in valley bottoms in the southern portion of the study area to >3000 m on mountain tops toward the north.

### Field Methods

We placed a survey grid over the seven mountain ranges in our study area, consisting of 453 5 × 5 km survey cells in the United States (US) and 237 10 × 10 km survey cells in Canada. Grid size in Canada was larger due to remoteness and lack of accessibility of this portion of the study area. We deployed ≥1 winter multi-species bait station per cell with a total of 734 bait stations deployed in 680 cells. Bait stations consisted of a bait (ungulate portion or frozen beaver [*Castor canadensis*]) attached to a tree, remote camera (all US stations and some Canada stations), and 12.30 caliber bronze rifle bore gun brushes (US) or barbed wire (Canada) positioned concentrically around the tree that acted as hair snags (see Robinson et al., 2017 for details). At Canadian stations, track surveys were completed at each bait station visit (see Kortello et al., 2019 for details).

### DNA Extraction, Microsatellite Amplifications, and mtDNA Sequencing

DNA extraction from hair samples and microsatellite amplification was performed using standard protocols (Paetkau, 2003; Kendall et al., 2009). We genotyped 241 marten individuals at 16 microsatellite loci (Ma-1, Ma-2, Ma-3, Ma-5, Ma-7, Ma-8, Ma-11, Ma-14, Ma-15, Ma-18, Ma-19 (Davis and Strobeck, 1998), Mer041, Mvis020 (Flemming et al., 1999), MP0055, MP0085, MP0227 (Jordan et al., 2007), plus an un-published ZFX/ZFY gender locus (primers w542: TTC CAG GCA GTA CCA AAC AG and w543: AGG AAA TCA TTC ATG AAT ATC ACT). We used GENEPOP 3.4 option 1 (Raymond and Rousset, 1995) to test each marker for a deficit of heterozygous genotypes. We produced 493 base pair sequences of the mitochondrial *cytochrome c oxidase I* (*COI*) for 175 individual marten (GenBank accession numbers MT578095 – MT578269). Macrogen Inc., produced sequence reads using PCR product we provided and primers we designed (forward 5′-CCTATTGATTCGCGCTGA-3′; reverse 5′-GTCTGTAAGTAGTATGGTAATGCC-3′).

### Phylogeny Estimation

To place our mtDNA data in geographic context we used published 493 base pair COI sequences (Colella et al., 2019) from 82 specimens (GenBank accession numbers MK320161 – MK320242). Sequence alignment was performed using Geneious 7.1.7 (Biomatters Ltd.) and the subsequent alignment and translation was checked by eye; no indels or stop codons were present. We then estimated the mtDNA gene tree under maximum parsimony (MP) and maximum likelihood (ML) optimization criteria using PAUP^∗^ (v. 4.0a, build 166; Swofford, 2003). Maximum Parsimony was performed using the heuristic search algorithm with random addition replicates, tree bisection-reconnection (TBR) branch swapping, and all sites and transformations were weighted equally. For the model-based ML analyses, we used the first (of 4) MP trees to evaluate models of nucleotide sequence evolution via the automodel command in PAUP^∗^ under the Bayesian Information Criterion (BIC) and decision theory (DT) (Minin et al., 2003). We then conducted heuristic searches under ML with the Kimura 2 parameter model (Kimura, 1980), TBR branch swapping, ten random-addition replicates, and a random starting tree. Nodal support for the ML tree was evaluated via bootstrap analysis with 500 pseudo-replicates (starting tree built using simple stepwise addition and NNI branch swapping) (Felsenstein, 1985).

### Population Genetic Analyses

We assessed genetic structure without *a priori* constraints using the clustering software STRUCTURE v2.3 (Pritchard et al., 2000). We performed 10 runs at each value of *K* from 1–5, with 250,000 MCMC iterations after a burn-in of 50,000, using the correlated allele frequencies and the admixture model.

We used GENEPOP 3.4 (Raymond and Rousset, 1995) to calculate observed (*H*_*o*_) and expected (*H*_*e*_) heterozygosity in each mountain range, estimate per locus *F*_*ST*_ values (Weir and Cockerham, 1984) between mountain ranges, and non-random associations of alleles within and between loci. We *a priori* defined populations by mountain range with the exception of the Central and South Selkirks, which we defined as two separate populations because they were separated by a large lake and low elevation valley with considerable human settlement. First, we estimated *F*_*ST*_ using 15 microsatellites (all except *Mvis020*). Second, to contextualize these observations, we used 9 of 11 markers used in a Canada-wide dataset (Kyle and Strobeck, 2003). We estimated contemporary effective population size (*N*_*e*_) using the linkage disequilibrium (i.e., non-random allele associations) method (Waples and Do, 2008) as implemented in both Ne Estimator v2 (Do et al., 2014) and LDNe (Waples and Do, 2008) at three levels of lowest allele frequency (0.01, 0.02, 0.05).

### Gene Flow Models

The phylogeny estimation and STRUCTURE analyses identified a population break in our study and we used this break to inform our gene flow analyses. We used GeneClass2 (Piry et al., 2004) to test all 48 southern individuals and the 75 northern individuals located closest to the break for evidence of ancestry from each side of the break (Paetkau et al., 1995, 2004).

We partitioned the entire dataset by lineage into northern (*M. americana*) and southern (*M. caurina*) populations and evaluated 3 putative models of gene flow and subsequently compared those models to investigate possible restrictions in the directionality of gene flow. We used the coalescent-based program migrate-n (Beerli and Felsenstein, 1999, 2001) to jointly estimate the mutation-scaled effective population size (θ = 4*N*_*e*_μ) and the mutation-scaled migration rate (*M* = *m*/μ; where *m* and μ are the migration and mutation rates, respectively), which can be used to estimate gene flow by θ ^∗^*M* = 4*N*_*e*_*m* where *N*_*e*_*m* is the number of migrants per generation. In model *M. americana* ⇄ *M. caurina*, gene flow is permitted in both directions between northern and southern populations. In model *M. americana* ⟶ *M. caurina*, gene flow is possible only in the southward direction while in model *M. americana* ⟵ *M. caurina*, gene flow is only in the northward direction. For each analysis in migrate-n, we used Bayesian inference for both microsatellite and mtDNA data. Starting values of θ and *M* were estimated with an *F*_*ST*_ calculation and the following uniform priors were specified: for microsatellites, θ, minimum = 0.0, maximum = 50.0, delta = 1.0 and *M*, minimum = 0.0, maximum = 500.0, delta = 50.0; for mtDNA, θ, minimum = 0.0, maximum = 0.1, delta = 0.001 and *M*, minimum = 0.0, maximum = 1000.0, delta = 100.0. Analyses were conducted using four replicates, four static-heated chains (1.0, 1.5, 3.0, 100,000.0) ran for 1,000,000 generations, sampling every 100, after 10,000 were discarded as burn-in. We inspected posterior distribution plots of estimated *M* and θ values (bin number = 1500) to assess convergence. We converted estimates of *M* and θ to the fraction of immigrants in population *i* that came from population *j* in the last generation via *Nm* = θ*_*i*_* ^∗^ *M*_*j* →_ *_*i*_* (this value was divided by 4 for the diploid, biparentally inherited microsatellites). In order to compare the different gene flow models, we obtained marginal likelihoods for each model (Kass and Raftery, 1995; Beerli and Palczewski, 2010), which can be used to evaluate multiple models when based on the same data using Bayes factors (Bloomquist et al., 2010). In the case of the 15 microsatellite loci, information from all loci was combined into a global estimate by Bezier approximation of the thermodynamic scores. The probability of a certain model was then obtained by dividing the exponent (on the base of e) log likelihoods by the sum of all exponent log likelihoods (Beerli and Palczewski, 2010).

### Spatial Patterns of Genetic Diversity

To capture potentially clinal genetic diversity patterns we used Spatial Genetic Diversity (sGD, Shirk and Cushman, 2011, 2014) to calculate local measures and map continuous gradients of genetic diversity across the study area. We used land cover and elevation to create a resistance surface for use in sGD. We expected less-forested habitat to be increasingly resistant to marten movement. Therefore, we assigned forest a resistance of 1 and non-forest areas were assigned incrementally higher resistance with increasing contrast to forest habitat (e.g., Vergara et al., 2017). The highest resistance value of 20 was given to water bodies, urban areas, and ice fields. Elevation resistance was defined using an inverted Gaussian function as has been done previously for marten in the study area (Wasserman et al., 2010). Also, similar to Wasserman et al. (2010) we used a dispersal distance of 15 km through average resistance values on the landscape to create a neighborhood search radius of 40,000 cost units to define genetic neighborhoods. We calculated allelic richness (*Ar*), observed heterozygosity (*H*_*o*_), inbreeding coefficient (*F*_*IS*_), and genetic neighborhood size (*NS*) in a 15 km diameter surrounding each sampled individual.

## Results

### Detections and Distribution

Marten were the most commonly detected mammal at bait stations and were well distributed across the study area with the exception of the Coeur d’Alene Mountains (CDA, Figure 1). We detected marten at only 12% of bait stations in the CDAs, but at 63% of other US bait stations.

### Phylogeny Estimation

**FIGURE 2:**
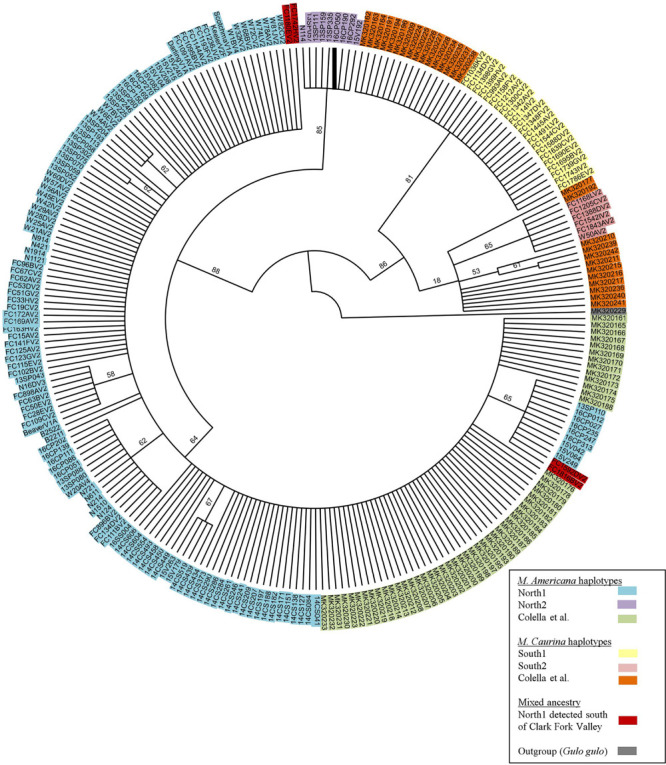
Bootstrap consensus tree of 493bp COI segment generated for this study and those of Colella et al. (2019).

Within the 493 COI nucleotides there are 10 concordant polymorphic sites that differentiate two haplotypes common to individuals from north of the Clark Fork Valley (North1 and North2) from two haplotypes common to marten south of Highway 200 (South1 and South2) (Figures 1, 2). Among the 143 COI sequences from north of Highway 200, 134 had the most common ‘North1’ haplotype while 9 (7 Purcells, 1 Valhalla, 1 South Selkirks) had a ‘North2’ variant that differed at three sites. Among the 32 sequences from south of the Clark Fork Valley, 22 individuals had a standard ‘South1’ haplotype and 6 (all from the Joe) had a variant ‘South2’ that differed at three sites. No southern haplotypes were detected north of the Clark Fork Valley but 4 southern individuals (3 Joe, 1 CDA) had the North1 haplotype (Figure 1). The North2 haplotype had a variant that differed at 2 non-diagnostic sites (94 and 268) but that also aligned with South1 and South2 at one diagnostic site (325). In the phylogeny (Figure 2), both North1 and North2 haplotypes grouped with sequences Colella et al. (2019) identified as *M. americana* with 88% bootstrap support. South1 and South2 grouped with sequences Colella et al. (2019) identified as *M. caurina* with 86% bootstrap support.

### Population Genetic Analyses

We produced genotypes for 16 microsatellite loci for 241 samples that assigned to 235 marten individuals. We removed the six repeat individual samples leaving us with 235 marten individuals (164 male, 61 female, and 10 unknown gender). Five individuals had incomplete genotypes, with a maximum of 2 loci missing per individual. Forty eight individuals were sampled south of the Clark Fork Valley and 187 to the north. Marker Mvis020 showed a large and highly significant (*P* < 0.0001) deficit of heterozygous genotypes. Of the 39 heterozygotes at *Mvis020* we had identified 37 as female. This suggests that *Mvis020* is on the X-chromosome (and that we misidentified 2 females as male), so we removed it from our population genetics analyses. None of the other 15 markers showed significant heterozygote deficits with a Bonferroni correction.

**FIGURE 3:**
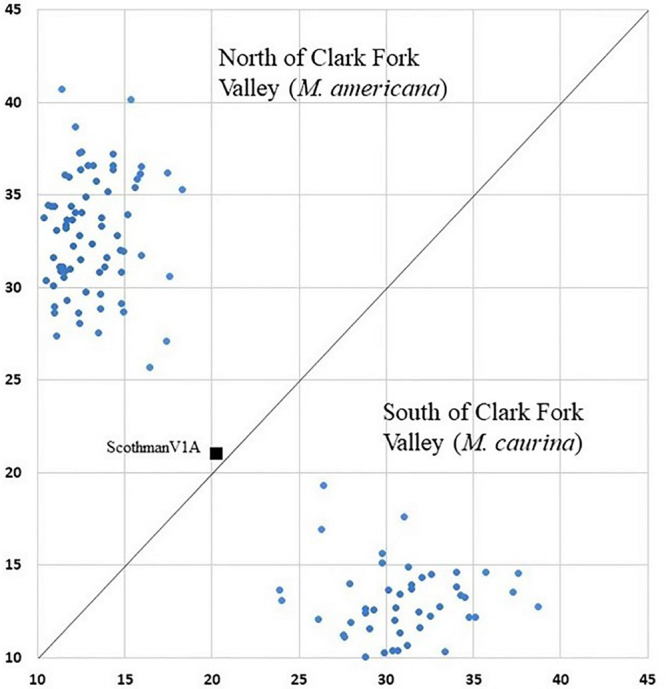
Geneclass assignment plot with mixed ancestry individual ScotchmanV1A (black square) shown between northern individual and southern individual clusters.

Genotypic differentiation (Genepop Option 3) was significant between all 21 pairs of mountain ranges. These results indicate that our population boundaries (valleys between mountain ranges) generally corresponded as genetic barriers to movement (Figure 3). After correcting for the number of tests (120 pairs of markers tested) we found no evidence of non-random associations of alleles between markers with a Bonferroni correction (“linkage disequilibrium”; Genepop Option 2).

*H*_*o*_ and *H*_*e*_ were similar across the study system with 15-locus averages ranging lowest in the Valhallas (*H_*o*_* = 0.51, *H_*e*_* = 0.54) to highest in the Central Selkirks (*H*_*o*_ = 0.60, *H*_*e*_ = 0.60). The Valhallas has the greatest heterozygote deficit (0.03) of any mountain range. The mean *H_*o*_-H_*e*_* deficit across all mountain ranges was 0.00, and averages of *H*_*o*_ and *H*_*e*_ across mountain ranges were and were similar for *M. americana* (*H*_*o*_ = 0.57, *H*_*e*_ = 0.58) and *M. caurina* (*H*_*o*_ = 0.57, *H*_*e*_ = 0.56) (Table 1).

**TABLE 1:** Observed heterozygosity (*H*_*o*_) and expected heterozygosity (*H*_*e*_) for each locus by mountain range, species, and full study area.

	Central selkirks			W Cabinets			Purcells			South selkirks			Valhallas			CDA			Joe			*M. americana*			*M. caurina*			Full study area		
Locus	N	He	Ho	N	He	Ho	N	He	Ho	N	He	Ho	N	He	Ho	N	He	Ho	N	He	Ho	He	Ho	Deficit	He	Ho	Deficit	He	Ho	Deficit
Ma-1	34	0.79	0.79	17	0.82	0.88	50	0.79	0.72	65	0.64	0.58	17	0.69	0.53	9	0.73	0.56	39	0.73	0.72	0.75	0.70	0.04	0.73	0.64	0.10	0.74	0.68	0.06
Ma-2	36	0.75	0.75	17	0.68	0.71	50	0.68	0.62	65	0.71	0.72	17	0.64	0.71	9	0.82	0.78	39	0.77	0.64	0.69	0.70	–0.01	0.80	0.71	0.09	0.72	0.70	0.02
Ma-3	36	0.03	0.03	17	0.00	0.00	50	0.18	0.16	65	0.34	0.28	17	0.06	0.06	9	0.54	0.56	39	0.55	0.49	0.12	0.10	0.02	0.55	0.52	0.02	0.24	0.22	0.02
Ma-5	36	0.69	0.67	17	0.71	0.71	50	0.79	0.78	65	0.63	0.65	17	0.64	0.59	9	0.86	0.78	39	0.81	0.69	0.69	0.68	0.02	0.84	0.74	0.10	0.73	0.69	0.04
Ma-8	36	0.76	0.75	17	0.79	0.88	50	0.74	0.80	65	0.75	0.78	17	0.68	0.76	9	0.69	0.89	39	0.71	0.72	0.74	0.80	–0.05	0.70	0.80	–0.10	0.73	0.80	–0.07
Ma-11	36	0.51	0.53	17	0.64	0.65	49	0.55	0.55	65	0.65	0.68	17	0.54	0.41	9	0.11	0.11	38	0.17	0.18	0.58	0.56	0.02	0.14	0.15	–0.01	0.45	0.44	0.01
Ma-14	36	0.69	0.69	17	0.85	0.82	50	0.84	0.82	65	0.78	0.80	17	0.77	0.82	9	0.60	0.56	39	0.51	0.51	0.79	0.79	–0.01	0.56	0.53	0.02	0.72	0.72	0.00
Ma-15	36	0.44	0.47	17	0.54	0.41	50	0.37	0.36	65	0.24	0.25	17	0.17	0.18	9	0.11	0.11	39	0.05	0.05	0.35	0.33	0.02	0.08	0.08	0.00	0.27	0.26	0.01
Ma-18	36	0.78	0.78	17	0.71	0.71	50	0.69	0.72	65	0.73	0.72	17	0.81	0.71	9	0.65	1.00	39	0.44	0.44	0.75	0.73	0.02	0.54	0.72	–0.17	0.69	0.72	–0.04
Ma-19	35	0.59	0.69	17	0.70	0.65	50	0.72	0.78	65	0.71	0.77	17	0.48	0.41	9	0.73	0.78	38	0.63	0.71	0.64	0.66	–0.02	0.68	0.74	–0.07	0.65	0.68	–0.03
Mer041	36	0.49	0.44	17	0.75	0.59	50	0.59	0.64	65	0.48	0.45	17	0.39	0.47	9	0.79	0.78	39	0.82	0.90	0.54	0.52	0.02	0.81	0.84	–0.03	0.62	0.61	0.01
Ma-7	36	0.62	0.56	17	0.44	0.29	50	0.35	0.28	65	0.52	0.55	17	0.51	0.53	9	0.76	0.78	39	0.67	0.74	0.49	0.44	0.04	0.72	0.76	–0.04	0.55	0.53	0.02
MP0055	36	0.65	0.61	17	0.61	0.59	50	0.34	0.34	65	0.38	0.34	17	0.61	0.53	9	0.00	0.00	39	0.03	0.03	0.52	0.48	0.04	0.01	0.01	0.00	0.37	0.35	0.03
MP0085	36	0.54	0.67	17	0.06	0.06	50	0.38	0.24	65	0.48	0.46	17	0.40	0.41	9	0.54	0.56	39	0.60	0.64	0.37	0.37	0.00	0.57	0.60	–0.03	0.43	0.43	–0.01
MP0227	36	0.69	0.56	17	0.74	0.76	50	0.70	0.76	65	0.62	0.51	17	0.68	0.59	9	0.67	0.78	39	0.64	0.77	0.68	0.64	0.05	0.65	0.77	–0.12	0.68	0.67	0.00
**Mean**		0.60	0.60		0.60	0.58		0.58	0.57		0.58	0.57		0.54	0.51		0.57	0.60		0.54	0.55	0.58	0.57	0.01	0.56	0.57	**−0.02**	**0.57**	**0.57**	**0.00**

**FIGURE 4:**
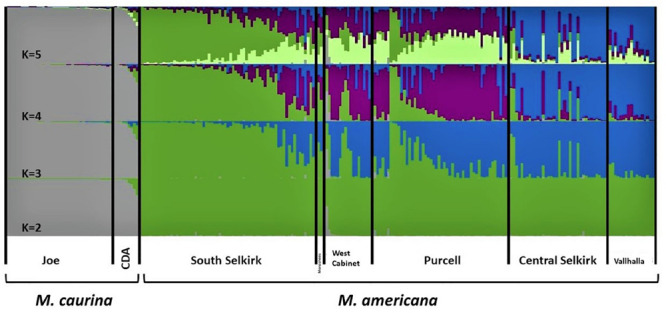
Apportioning of ancestry for 235 *M. americana* and *M. caurina* in the highest likelihood runs in STRUCTURE at *K* = 2 (bottom panel) through 5 (top panel).

**FIGURE 5:**
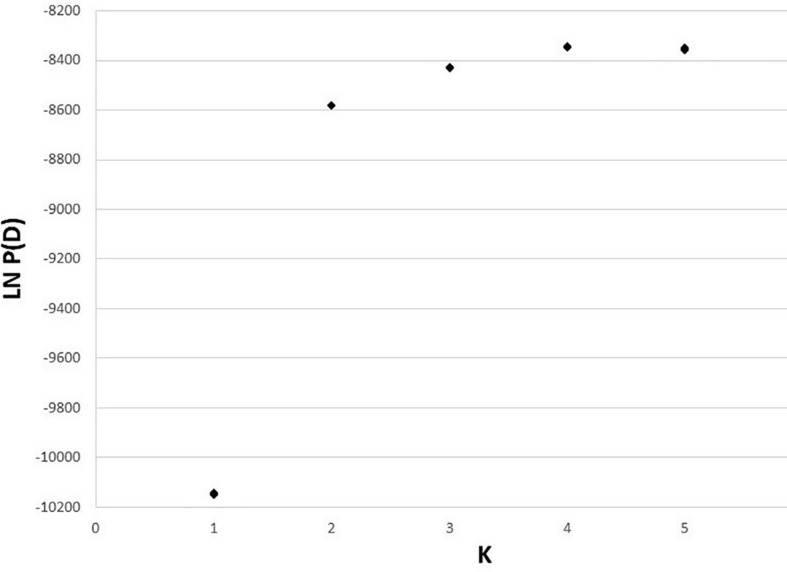
Estimated log likelihood of K for 50 runs of STRUCTURE (10 each at *K* = 1–5).

The 10 STRUCTURE runs at each value of *K* converged strongly at each *K*, with likelihoods peaking at *K* = 4. The largest lnP(D) increase (1,600) occurred at *K* = 2 identifying a major split at the Clark Fork Valley. Smaller, less discrete, genetic breaks were identified at *K* = 3 and *K* = 4, most notably identifying a sharp split between the South Selkirks and Valhallas. The new split introduced between *K* = 4 and *K* = 5 divided 1 of the 4 earlier ancestries in such a way that no individual’s ancestry could be traced predominantly to either new group, thus failing to identify evidence of five genetic populations within the study region (Figures 4, 5).

The *K* = 2 model assigned mixed ancestry to several individuals immediately on either side of the Clark Fork. Most notably, the Cabinet sample ScotchmanV1A was estimated to have roughly equal ancestry north and south of the river, suggesting that it may have one parent from either side (Figure 1). Four of the 9 CDA individuals had substantial estimates of northern ancestry, in the range expected of second or third generation hybrids (3/4 or 7/8 southern ancestry). There was no evidence of recent mixed ancestry in the Joe, where a mean of 99% and minimum of 95% of ancestry was assigned to the southern group (gray in Figure 4).

The 15-locus analysis produced *F*_*ST*_ values similar to (range 0.03–0.32, mean = 0.17), but slightly higher than the 9-locus analysis (range 0.03–0.30, mean = 0.15; Table 2). *F*_*ST*_ values confirmed the Clark Fork Valley split identified by Structure at *K* = 2. Values across the Clark Fork Valley range from 0.21 to 0.32 with smaller values among northern (0.03–0.09) and southern (0.03) mountain ranges.

**TABLE 2:** *F*_*ST*_ values between mountain ranges.

Mountain Range	CDA	Joe	Purcell	Central Selkirk	South Selkrik	Valhalla	W Cabinet
CDA		0.03	0.24	0.24	0.21	0.26	0.21
Joe	0.03		0.29	0.29	0.25	0.30	0.24
Purcell	0.28	0.32		0.05	0.05	0.06	0.04
Central Selkrik	0.26	0.31	0.04		0.05	0.03	0.06
South Selkirk	0.26	0.31	0.04	0.05		0.08	0.03
Valhalla	0.28	0.32	0.06	0.03	0.07		0.06
W Cabinet	0.26	0.30	0.05	0.06	0.06	0.09	

*Estimates above the diagonal are based on 9 of the 11 markers used by Kyle and Strobeck (2003). Estimated below the diagonal are based on all 15 microsatellites analyzed for this study.*

Both estimation methods for contemporary *Ne* (NeEstimator and LDNe) and the three allele frequencies used produced similar results (Table 3). Although confidence decreased with lower allele frequencies used, *N*_*e*_ values remained generally similar across tests for most mountain ranges. The CDA (*Ne* = 31.2) and West Cabinets (*Ne* = 39.8–45.8) had the lowest and most consistent *Ne* values across allele frequencies. The remaining mountain ranges had higher and less consistent *Ne* values across allele frequencies.

**TABLE 3:** Estimates of contemporary effective population size (*N*_*e*_) and 95% confidence intervals obtained from individuals genotyped at 15 microsatellite loci by mountain range.

			Effective population size (Ne)		
Mountain Range			Lowest allele frequency used		
	Method	*n*	0.05	0.02	0.01
CDA	LDNe*	9	31.2 (10.5-inf)	31.2 (10.5-inf)	31.2 (10.5-inf)
	NeEst**	9	31.2 (10.5-inf)	31.2 (10.5-inf)	31.2 (10.5-inf)
Joe	LDNe	39	138.6 (63.4-inf)	395.9 (116.8-inf)	763.9 (153.2-inf)
	NeEst	39	138.2 (63.3-inf)	393.1 (116.6-inf)	754.4 (152.8-inf)
W Cabinet	LDNe	17	45.8 (21.8−474.4)	39.8 (20.9−158.7)	39.8 (20.9−158.7)
	NeEst	17	45.8 (21.8−474.4)	39.8 (20.9−158.7)	39.8 (20.9−158.7)
Purcell	LDNe	50	176.6 (85.6−2242.1)	169.4 (90.8−725.4)	214.4 (107.9−1817.1)
	NeEst	50	176.6 (85.6−2236.5)	169.2 (90.7−723.0)	214.1 (107.9−1796.0)
Central Selkirk	LDNe	36	155.1 (65.8-inf)	117.2 (61.8−547.0)	81.4 (50.4−176.8)
	NeEst	36	152.8 (65.3-inf)	115.8 (61.3−516.9)	80.6 (50.1−173.4)
South Selkirk	LDNe	65	97.2 (63.4−180.0)	159.0 (93.9−407.5)	183.4 (105.2−536.5)
	NeEst	65	97.2 (63.4−180.0)	159.0 (93.9−407.5)	183.4 (105.2−536.5)
Valhalla	LDNe	17	82.6 (24.1-inf)	872.7 (49.0-inf)	872.7 (49.0-inf)
	NeEst	17	82.6 (24.1-inf)	872.7 (49.0-inf)	872.7 (49.0-inf)

Estimates were done using both *LDNe and **NeEstimator and three sets of lowest allele frequency used (0.05, 0.02, 0.01).

### Gene Flow Models

Of the 123 individuals tested in GeneClass2 for ancestry from either side of the break, only ScotchmanV1A (Figure 1) had a genotype that was inconsistent with pure ancestry on the side of the Clark Fork where it was sampled (*p* < 0.0001) (Figure 3).

The evaluation of three models tested (*M. americana* ⇄ *M. caurina*, *M. americana* ⟶ *M. caurina*, and *M. americana* ⟵ *M. caurina*) showed evidence of gene flow occurring under each model but gave most support (probability > 0.99) to *M. americana* ⟶ *M. caurina* for both mtDNA and microsatellite data. These results indicate gene flow occurring primarily, but not exclusively, from north to south. Estimates of the fraction of immigrants were relatively low for all models. The *M. americana* ⟶ *M. caurina*, model estimated the fraction of immigrants at 1.09 (0.031–5.15; mtDNA) and 0.89 (0.0–3.79; microsatellites), *M. americana* ⇄ *M. caurina* at 1.18 (0.03–5.7, mtDNA) and 1.99 (0.0–6.89 microsatellites), and *M. americana* ⟵ *M. caurina* at 1.05 (0.01–4.8, mtDNA) and 1.82 (0.0–6.04, microsatellites).

### Spatial Patterns of Genetic Diversity

**FIGURE 6:**
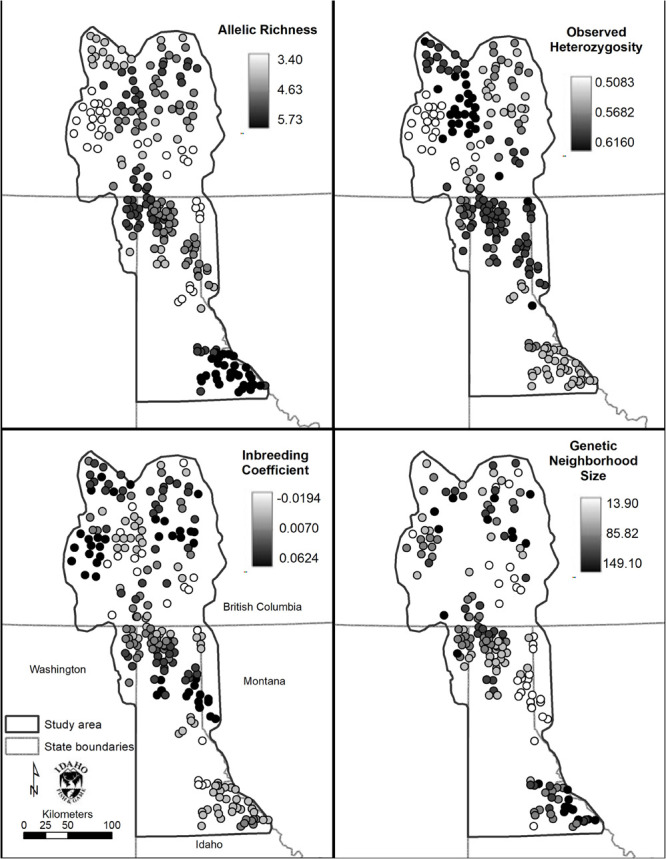
Spatial genetic distance (sGD) analysis results. Clockwise from top left: Allelic richness (*Ar*), observed heterozygosity (*Ho*), inbreeding coefficient (*F*_*IS*_), and genetic neighborhood size (*NS*).

Allelic richness was highest in the Joe (mean = 5.42) followed by South (mean = 4.66) and Central (mean = 4.62) Selkirks (Table 4). The Joe samples all showed high levels of allelic richness while the South and Central Selkirk ranges showed clustering of high allelic richness in the central portions of each range with a notable drop in allelic richness toward the northwestern portion of the Central Selkirks (Figure 6). The lowest allelic richness was in the CDAs (mean = 3.81) and Valhallas (mean = 3.84).

**TABLE 4:** Mean values by mountain range of clinal allelic richness (*A*), observed heterozygosity (*H*_*o*_), inbreeding coefficient (*F*_*IS*_), and genetic neighborhood size (*NS*) of the sGD analysis.

Range	*A*	*H*_*o*_	*F*_*IS*_	*NS*
CDA	3.81	0.56	–0.02	31.20
Joe	5.42	0.55	–0.01	106.62
W Cabinet	4.63	0.58	0.03	48.81
Purcell	4.37	0.57	0.01	79.17
Central Selkirk	4.62	0.60	0.00	99.76
South Selkirk	4.66	0.57	0.00	88.83
Valhalla	3.84	0.52	0.04	85.27

Observed heterozygosity had little variation and values were similar (0.52–0.60) to those calculated in GenePop based on *a priori* population definitions. *F*_*IS*_ values ranged from −0.02 to 0.04 with mountain ranges averaging lowest in the CDA (mean *F*_*IS*_ = −0.02) and Joe (mean *F*_*IS*_ = −0.01) and highest in the West Cabinets (mean *F*_*IS*_ = 0.03) and Valhallas (mean *F*_*IS*_ = 0.04). Genetic neighborhood sizes averaged lowest for the CDAs (mean *NS* = 31.20) and West Cabinets (mean *NS* = 48.81), was highest in the Joe (mean *NS* = 106.62), and there was a cluster of high NS values in the US portion of the South Selkirks (Figure 6 and Table 4).

## Discussion

### Species Fracture Zone and Taxonomic Alignment

We observed a larger genetic break across the approximately 5 km wide Clark Fork Valley (*F*_*ST*_ = 0.21–0.30) than was observed by Kyle and Strobeck (2003) between Golden, British Columbia (northern portion of our study area) and the island of Newfoundland (*F*_*ST*_ = 0.21, Kyle and Strobeck, 2003). In addition to lying 4,000 km to the east, Newfoundland has not had a terrestrial connection with the rest of North America since the end of the Pleistocene glaciations (Pielou, 2008). During the last glacial maximum, approximately 20,000 years before present (Clark et al., 2009), the Clark Fork Valley along with the CDA Mountains were part of the large, 300 km long, Lake Missoula (Pielou, 2008). North of Lake Missoula, our study area was largely engulfed by the Cordilleran Ice Sheet (Pielou, 2008) with the remaining southern portion of our study area being ice free and part of the Pacific Northwest Refugium (Shafer et al., 2010). *M. caurina* are among the 150 known species or species complexes that have disjunct mesic forest inland and coastal populations in Pacific Northwest (Carstens et al., 2004) due to desertification of the Columbia Plateau (Shafer et al., 2010). During last glacial maximum many species persisted in ice free Pacific Northwest refugia (e.g., Lucid and Cook, 2004) while maintaining genetic connectivity to populations persisting in coastal refugia (Sawyer et al., 2019). The fossil record indicates marten persisted disjunctly during last glacial maximum in western and eastern North America with the eastern group expanding west from the late Pleistocene to late Holocene (Graham and Graham, 1994). Our findings are consistent with the hypothesis (Stone et al., 2002) that *M caurina* persisted in ice free Pacific Northwest refugia while *M. americana* persisted in eastern refugia and expanded west as glaciers retreated.

The only individual we identified as likely to have a parent from either side of the break (ScotchmanV1A, Figure 1) was sampled north of the Clark Fork Valley, 8 km from the river itself. However, we identified northern mitochondrial haplotypes in 4 of 32 individuals sampled south of the break, including 3 individuals from the Joe, relatively distant from the break, whereas we observed no southern haplotypes to the north. Colella et al. (2019) found a similar asymmetry in the distribution of mitochondrial haplotypes, suggesting that gene flow from north to south has been taking place for some time. The preponderance of evidence suggests *M. caurina* likely occupied the ice-free Joe during last glacial maximum and *M. americana* colonized the northern portion of our study area from the east as glaciers retreated. Marten would not have been able to establish populations in the CDA during last glacial maximum due to the periodic Lake Missoula floods but, after a terrestrial connection was available, gene flow likely initiated and was predominate from *M. americana* (north) to *M. caurina* (south). This glacial history has lead us to today where, for practical management purposes, the Clark Fork Valley serves as a geographic boundary between the two lineages with the *M. caurina* lineage occurring to the south and *M. americana* lineage occurring to the north of the valley.

### Effective Population Size

Effective population sizes (*N*_*e*_), or number of breeding individuals, are relatively modest considering the size of the mountain ranges. Estimates of clinal genetic neighborhood sizes (*NS*), also number of breeding individuals, range from 13.9 to 149.1 within a 15 km radius of individual marten. Estimation of clinal *NS* eliminates the assumption of panmixia from breeding individual estimates and has been shown to estimate larger numbers of breeding individuals than *N*_*e*_ (Shirk and Cushman, 2014). However, developing global estimates from *NS* values has limited feasibility and since being introduced as a concept (Wright, 1946), *NS* has been used sparingly by researchers leaving few examples from which to extrapolate relevance. In contrast, *N*_*e*_ is a widely accepted as a variable to quantify a population’s capacity to resist loss of genetic diversity through drift (Shirk and Cushman, 2014). Therefore, it was important to use a variety of techniques to provide opportunities for context and comparison of results.

We used seven (*NS* plus 2 software program and 3 allele frequencies for *N*_*e*_) techniques to estimate number of breeding animals for each mountain range. Although our *N*_*e*_ confidence intervals were generally quite large, comparing them to the *NS* estimates show similar general patterns of abundance across the study area with moderate numbers (hundreds) estimated for most mountain ranges and depressed numbers in the CDAs (*N*_*e*_ = 31.2, mean *NS* = 31.2) and West Cabinets (*N*_*e*_ = 39.8–45.8, mean *NS* = 48.81).

### Inter-Specific Gene Flow

The individual of recent mixed ancestry is direct evidence that contemporary gene is occurring. At first glance, this appears contrary to the Migrate results showing dominate gene flow from north (*M. americana*) to south (*M. caurina*). However, all models predict less than one migrant per generation across the break. We suspect our sampling window was too short and sample sizes too small to detect the few first generation hybrids that may be on the landscape and that the longer term process is predominantly north to south, as the Migrate models suggest. This asymmetry may reflect a higher likelihood for *M. americana* individuals to disperse due to proximate local population conditions or stochastic events.

### *M. americana* Genetic Structure North of the Fracture Zone

The Valhallas stand out as the most genetically distinct *M. americana* mountain range with the highest northern *F*_*ST*_ values. Compared to Valhalla, West Cabinet (*F*_*ST*_ = 0.09) values are higher than South Selkirk values (*F*_*ST*_ = 0.03) showing an expected lowering in value with reduced distance. However, the adjacent South Selkirks (*F*_*ST*_ = 0.07) show a relatively high level of genetic disjunct with the Valhallas. *N*_*e*_ estimates varied widely for this range (82.6–872.7) but the relatively low mean allelic diversity (3.81) along the highest mean *F*_*IS*_ value (0.04) in the study area suggest the lower *N*_*e*_ estimates may be more reflective of reality.

The West Cabinets are surprisingly disjunct (*F*_*ST*_ = 0.06) from the South Selkirks given the MacArthur Wildlife Corridor purportedly connects the two ranges (Davidson, 2003). The Purcell-West Cabinet *F*_*ST*_ value (0.05) is slightly lower despite no “official” wildlife corridor connecting them to the West Cabinets. Although modeling work has suggested this corridor is important for grizzly bear and wolverine (*Gulo gulo*) movement (Cushman et al., 2006; Schwartz et al., 2009), field studies have provided suggestive evidence that fisher (*Pekania pennanti*) may not use the corridor (Lucid et al., 2019). The high level of marten genetic disjunct casts further doubt on the permeability MacArthur Wildlife Corridor by mammals.

In our study area, *M. americana* avoid crossing un-forested areas (Cushman et al., 2011, but see Mowat, 2006) and gene flow is primarily a function of elevation (Wasserman et al., 2010). That the mean detection elevation for *M. americana* in the US portion of our study area was 1,467 m (Lucid et al., 2016) is consistent with the Wasserman et al. (2010) finding of a minimum resistance to gene flow at 1,500 m. This result does not apply to marten continentally as marten are broadly distributed across low elevation boreal forests in Canada (Kyle and Strobeck, 2003), rather this result is limited to our study area and, perhaps, other mountainous portions of marten range of similar latitude. Recognizing climate change may shift this ideal elevation upward suggests marten corridor conservation efforts in our study area may be most effective at elevations at or higher than 1,500 m.

### Genetic Structure Along the Fracture Zone

The lowest population estimates in the study area occur along the fracture zone in the CDAs (*Ne* and *NS* = 31.2) and West Cabinets (*Ne* = 39.8–45.8, mean *NS* = 48.81). *F*_*IS*_ values show disparity, however, with the lowest study area values in the CDAs (mean *F*_*IS*_ = −0.02) and the second highest values in the West Cabinets (mean *F*_*IS*_ = 0.03). Although suggestive of a depressed West Cabinet and genetically robust CDA population, the interpretation of positive *F*_*IS*_ values can be less than straight forward. First, the signal created by null alleles, unrecognized population structure within mountain ranges, or a Wahlund effect (Wahlund, 1928) created by recent immigration could all contribute to increased *F*_*IS*_ estimates. Second, the genetic break across the Clark Fork Valley could lead to differential detectability of Wahlund effects. In a scenario where individuals move between populations with a history of high connectivity (i.e., low *F*_*ST*_), immigrants have genotypes that are consistent with the population into which they move, and thus their addition to the new population does not create a departure from Hardy-Weinberg expectation. By contrast, when immigrants move between populations with a history of strong isolation (i.e., high *Fst*, such as the Clark Fork Valley), they have genotypes that are inconsistent with origins in their destination population, and thus create a detectable Wahlund effect. The difference between these scenarios is not the current rate of movement, but the historical rate. Given the possibility of such an artifact, we cannot rule out that the identification of relatively high (but statistically non-significant) *F*_*IS*_ in the West Cabinets reflects greater historical separation, rather than contemporary differences in population processes relative to other mountain ranges that we sampled.

Low numbers of marten in the CDAs are supported both by low field survey detection along with low *N*_*e*_ and *NS* estimates. Therefore, the negative *F*_*IS*_ values are most likely a Wahuld effect as described in scenario 2 above. Although adjacent areas have been modelled as high quality marten habitat (i.e., Wasserman et al., 2012b), the relatively low elevation CDAs may provide lower quality habitat. Assuming relatively low marten density in the West Cabinets is the correct interpretation of the data, it could plausibly be driven by competition or predation by fisher, which are relatively abundant in the West Cabinets but largely absent from the rest of the study area (Lucid et al., 2019). Marten are currently managed as a single biological unit, *M. americana*, across the state of Idaho with unlimited state-wide harvest (Idaho Department of Fish and Game (IDFG), 2019). Recognizing the statistical imperfections of our results, the prudent management approach would be to re-evaluate harvest regulations in the CDAs and West Cabinets.

### *M. caurina* Genetic Structure South of the Fracture Zone

The Saint Joe Mountain Range had low *F*_*IS*_ values (mean = −0.01), the highest allelic richness (mean = 5.42), and some of the highest population estimates in the study area (mean *NS* = 106.62, *N*_*e*_ 138.6–763.9). These metrics suggest the Joe hosts a relatively large and genetically robust marten population. Our *M. cqurina* sample size (*n* = 48) is lower than *M americana* (*n* = 187) primarily due to the low population size in the CDAs and that the geographic area surveyed is smaller in the southern portion of our study area. Future work to determine how the Joe population is structured with other *M. caurina* populations to the east and south would be a valuable extension of this study.

## Conclusion

### Conservation and Management Implications

We identified genetic exchange across a strong genetic break that likely has roots in Pleistocene events, leading to the possibility of substantial local adaptation on either side of the break. Hybridization has been proposed as a conservation problem, particularly in areas where marten augmentation has occurred, with a detrimental effect to *M. caurina* (Colella et al., 2019). However, the natural erosion of genetic barriers between lineages lead us to recommend natural hybridization not be considered a conservation or management problem within our study area.

The strength of the genetic break that we identified shows that historical connectivity across the Clark Fork Valley has been low enough to produce more or less complete demographic independence: the trajectory of these populations will depend to a much greater extent on local birth and death than on immigration or emigration across the Clark Fork Valley. On this basis we suggest that these populations, which we identify as *M. americana* and *M. caurina*, should be treated as separate entities for monitoring programs and management decisions such as developing harvest recommendations.

While there is little doubt that historical connectivity across the Clark Fork Valley has been low, we identified evidence of interbreeding across this break in several individuals sampled near the boundary. This raises the possibility of a recent increase in exchange between highly distinct genetic groups, which may have impacts on fitness that are relevant to management. Genetic assessment of individual ancestry is uniquely suited to non-equilibrium scenarios of suddenly increased connectivity (Paetkau et al., 2009). We therefore recommend ongoing sampling within a band spanning perhaps 20 km on either side of the contact zone, combined with genetic assessment of ancestry to identify individuals likely to have dispersed across the Clark Fork Valley, as well as their offspring (F1s). Ideally, if a number of F1 individuals are detected, it would be possible to conduct more detailed assessments of survival and reproductive output of such individuals relative to individuals whose recent ancestry is from a single side of the genetic break.

In the meantime, we recommend that augmentation or translocation efforts should move marten only from areas with robust local populations from the same genetic group. Prior to augmentations in areas where marten density may be low, such as the CDAs and West Cabinets, in depth study should be undertaken to determine reasons behind depressed populations. Furthermore, harvest in these ranges should be more closely monitored and harvest limits considered.

Long term persistence of marten populations in our study area will be dependent on the maintenance of at least some gene flow amongst mountain ranges. Although we would not dissuade corridor conservation at any elevation, marten in the region that we studied would benefit most from corridor conservation efforts focused on areas of elevation 1,500 m or higher. Monitoring marten population genetic structure over time will be a crucial aspect in evaluating effectiveness of these measures and, as we have demonstrated, inclusion of marten into regional multi-taxa survey programs is an effective way to develop hair and genetic data collections.

## Data Availability Statement

The datasets presented in this study are publicly available. The raw microsatellite data are available in the Supplementary Material section. The COI sequence data are available at https://www.ncbi.nlm.nig.gov/genbank (accession numbers MT578095-MT578269).

## Ethics Statement

Ethical review and approval was not required for the animal study because all samples were collected with non-invasive methods. Author institutions do not require approval. However, we do follow the ethics policy of the American Society of Mammalogists.

## Author Contributions

ML conceived the overall study design, coordinated the data analysis, and wrote the manuscript. ML, LR, and SE conceived of the field survey design and oversaw the sample collection and database management in the United States. GM, DH, and AK conceived of field survey design and oversaw sample collection and database management in the Canada. DP oversaw and SG performed the laboratory analysis. SC, SG, JS, AR, and DP analyzed the data. LS contributed to the study design conception, preparation of spatial datasets, interpretation of the data, and the figure preparation. All authors read and approved the final manuscript.

## Conflict of Interest

DH was employed by the Seepanee Ecological Consulting Company. AK was employeed by the Grylloblatta Ecological Consulting Company. DP and SG were employed by the company Wildlife Genetics International. LR was employed but they Idaho Department of Fish and Game during the course of this research. However, LR now is employeed by the Rainforest Ecological Consulting Company. The remaining authors declare that the research was conducted in the absence of any commercial or financial relationships that could be construed as a potential conflict of interest.
